# Lavender (*Lavandula stoechas L.)* essential oils attenuate hyperglycemia and protect against oxidative stress in alloxan-induced diabetic rats

**DOI:** 10.1186/1476-511X-12-189

**Published:** 2013-12-28

**Authors:** Hichem Sebai, Slimen Selmi, Kais Rtibi, Abdelaziz Souli, Najoua Gharbi, Mohsen Sakly

**Affiliations:** 1 Laboratoire de Physiologie Intégrée, Faculté des Sciences de Bizerte, 7021 Zarzouna, Tunisia; 2 Département des Sciences de la Vie, Laboratore de Physiologie Animale, Faculté des Sciences de Tunis, Tunis 1060, Tunisia; 3 Laboratoire de Nutrition et Physiologie Animale - Institut Supérieur de Biotechnologie de Béja, Avenue Habib Bourguiba - B.P., 382-9000 Béja, Tunisia

**Keywords:** *Lavandula Stoechas*, Alloxan, Rat, Essential oils, Antioxidant, Antidiabetic

## Abstract

**Background:**

The present study described the phytochemical profile of *Lavandula stoechas* essential oils, collected in the area of Ain-Draham (North-West of Tunisia), as well as their protective effects against alloxan-induced diabetes and oxidative stress in rat.

**Methods:**

Essential oils samples were obtained from the aerial parts of the plant by hydrodistillation and analyzed by GC–MS. Rats were divided into four groups: Healthy Control (HC); Diabetic Control (DC); Healthy + Essential Oils (H + EO) and Diabetic + Essential Oils (D + EO).

Antidiabetic and antioxidant activities were evaluated after subacute intraperitoneally injection of *Lavandula stoechas* essential oils (50 mg/kg *b.w., i.p.*) to rats during 15 days.

**Results:**

The principal compounds detected are: D-Fenchone (29.28%), α-pinene (23.18%), Camphor (15.97%), Camphene (7.83%), Eucapur (3.29%), Limonene, (2.71%) Linalool, (2.01%) Endobornyl Acetate (1.03%). The essential oils also contained smaller percentages of Tricyclene, Cymene, Delta-Cadinene, Selina-3,7(11)-diene. Furthermore, we found that *Lavandula stoechas* essential oils significantly protected against the increase of blood glucose as well as the decrease of antioxidant enzyme activities induced by aloxan treatment. Subacute essential oils treatment induced a decrease of lipoperoxidation as well as an increase of antioxidant enzyme activities.

**Conclusions:**

These findings suggested that *lavandula stoechas* essential oils protected against diabetes and oxidative stress induced by alloxan treatment. These effects are in partly due to its potent antioxidant properties.

## Introduction

The Mediterranean diet has been credited with many beneficial effects in the prevention and treatment of cardiovascular diseases [1], including improvement of lipid profiles, reduction in blood pressure, insulin resistance, blood glucose concentration and inflammatory biomarkers [2]. Oxidative stress is believed to be a primary factor in various diseases as well as in the normal process of aging [3,4]. Free radicals and reactive oxygen species (ROS) are well known as inducers of cellular and tissue pathogenesis leading to several human diseases such as cancer, inflammatory disorders, atherosclerosis and cardiovascular diseases [5,6]. Cardiovascular diseases are the most common cause of death in the industrialized countries [7]. Many epidemiological and experimental studies have shown that phenolic compounds intake is inversely correlated with atherosclerosis development and related cardiovascular events [8-10]. The beneficial effect of polyphenols is associated with a multitude of biological activities, including antioxidant and free radical-scavenging properties, anti-platelet aggregation and inhibition of vascular smooth muscle cell proliferation. These observations might explain their cardiovascular protective properties [11]. On the other hand, it is now established that hyperlipidaemia represents a major risk factor for the premature development of atherosclerosis and its cardiovascular complications [12]. A logical strategy to prevent against atherosclerosis and reduce the incidence of cardiovascular disease events is to target the hyperlipidemia and oxidative stress by diet and/or drug intervention.Lavender (*Lavandula stoechas L.*) is a medicinal plant largely used in the Tunisian traditional medicine. In fact, this plant is known to protect against headaches, depression and diabetes [13,14]. *Lavandula stoechas* essential oils are rich in monoterpenes and employed for its antimicrobial, antifungal and carminative properties as well as its cosmetic purposes [14-16]. Its leaves and stems are used to prepare decoctions against rheumatism, chill and digestive system diseases [17]. Furthermore, Lavender essential oils are advocated for their use as an antibacterial agent in both early and modern aromatherapy texts [18,19].

Lavender has been extensively phytochemically studied, with limited work on pharmacological aspects and is used by traditional healers for various diseases of the central nervous system, like epilepsy and migraine [20]. It is also used in folk medicine, as an antispasmodic in colic pain and has analgesic, tranquillizer and antiseptic effects [20-23]. Lavender extracts have also positive effects on wound, urinal infections, cardiac diseases and eczema [22]. This species has been also shown to reduce blood sugar levels [13].

In the present study, we studied the protective effect of the *lavandula stoechas* essential oils against diabetes and oxidative stress induced by alloxan treatment in rat. We also examined their hepatoprotective and nephroprotective effects.

## Materials and methods

### Chemicals

Alloxan monohydrate, 5,5-dithio bis(2-nitrobenzoic acid) (DTNB), trichloroacetic acid (TCA), KOH, ethanol, ether, bovine serum albumin, orthophosphoric acid 85%, and NaCl were obtained from Sigma-Aldrich Co. (Germany).

### Plant collection

*Lavandula stoechas* (*L. stoechas*) aerial parts were collected in March, 2010 from the area of Ain-Draham (North-West of Tunisia) and identified by the laboratory of taxonomy in the Faculty of Sciences of Tunis (FST)-Tunisia. The Voucher specimens (No. L101) have been deposited with the herbarium of the Higher Institute of Biotechnology of Béja and also in Department of Biological Sciences, Faculty of Science, Tunisia.

### Plant extracts preparation

The dried aerial parts were submitted to hydrodistillation for 3 hours using Clevenger type apparatus, according to the European Pharmacopoeia (1996). Briefly, the plant was immersed in water and heated to boiling, after which the essential oils were evaporated together with water vapour and finally collected in a condenser. The distillate was isolated and dried over anhydrous sodium sulfate. The oils farctions were stored at 4°C until analysis by GC–MS.

### Free radical-scavenging activity on DPPH

The antioxidant capacity of the Lavender essential oils was performed using 2,2-diphenyl-1-picrylhydrazyl (DPPH) radical-scavenging activity as previously described by Grzegorczyk et al. [24]. Briefly, Various concentrations of the essential oils (20, 50, 100, 150, and 200 μg/ml) were added to 1 ml of 0.1 mM methanol solution of DPPH and incubated at 27°C during 30 min. The absorbance of the sample was measured at 517 nm. DPPH radical-scavenging activity (RSA), expressed as percentage was calculated using the following formula:

$$\mathrm{RSA}\left(\%\right)=\frac{A_{\mathrm{DPPH}}-\left(A_{\mathrm{sample}}-A_{\mathrm{control}}\right)}{A_{\mathrm{DPPH}}}\times 100$$

Ascorbic acid was used as a reference molecule in the same concentration as the test extract.

All the analyses were done in triplicate. The efficacy concentration 50 (EC50) value was defined as the concentration (in μg/ml) of the compound required to scavenge 50% of the DPPH radical.

### Gas chromatography–mass spectrometry (GC-MS)

The essential oils of *L. Stoechas* were subjected to GC-MS analysis using Trace GC ULTRA/Polaris Q (GC-MS, Thermo Electron). The column was a VB-5 (5% phenyl/95% dimethylpolysiloxane) with film thickness of 0.25 μm, a length of 30 m and an internal diameter of 0.25 μm helium was used as carrier gas. The GC oven temperature was kept at 50°C for 5 min and programmed to 250°C for 3 min at rate of 4°C/min and programmed to 300°C at rate of 25°C/min. The injector temperature was set at 250°C. Split flow was adjusted at 50 mL/min. MS were taken at 70 eV. Mass range was from uma 20 to 350. A library search was carried out using the “Wiley GC/MS Library”, Nist and Pmw. The sample was dissolved in Hexane.

### Animals and treatment

Adult male Wistar rats (weighing 220–230 g, 15 weeks old and housed five per cage) were purchased from SIPHAT (Tunis, Tunisia) and used in accordance with the local ethic committee of Tunis University for use and care of animals in conformity with the NIH recommendations. The animals were housed in standard cages (40× 28 ×16 cm) under controlled conditions: 12:12-h light–dark, 20–22°C, food and water are *ad libitum*. After diabetes induction, rats were divided into 4 groups of 12 animals each:

Group I: non-diabetic rat (control) treated with NaCl (0.9%, *i.p.*) during 15 days.

Group II: diabetic rat (alloxan, 220 mg/kg *b.w.*) treated with NaCl (0.9%) during 15 days.

Group III: non-diabetic rat treated with essential oils (50 mg/kg *b.w., i.p.*) during 15 consecutive days.

Group IV: diabetic rat (alloxan, 220 mg/kg *b.w. i.p.*) treated with essential oils (50 mg/kg *b.w., i.p.*) during 15 consecutive days.

Twenty hours after the last injection, animals were sacrificed, blood was collected in heparinized tubes. After centrifugation at 3 000 g for 15 min, plasma was processed for biochemical parameter determinations. The liver and the kidney were rapidly excised and homogenized in phosphate buffer saline. After centrifugation at 10 000 g for 10 min at 4°C, supernatants were used for biochemical determination of protein, -SH groups, MDA and antioxidant enzyme activities.

### Induction of experimental diabetes

Experimental diabetes was induced in 12 h fasted rats by single *i.p.* injection of alloxan (220 mg/kg body weight) [25] dissolved in citrate buffer (100 mM, pH 4.5). To prevent fatal hypoglycaemia as a result of massive pancreatic insulin release, the rats were supplied with 10% glucose solution after 6 hours of alloxan administration for the next 24 hours. After seven days, rats with glycemia ≥14 mM were selected for the experiment.

### Lipid peroxidation

Lipid peroxidation was detected by the determination of MDA production determined by the method of Begue and Aust [26]. Briefly, homogenates of liver and kidney were centrifuged at 1000 g for 10 min at 4°C to sediment cell debris and mitochondrial samples. Supernatants were suspended in PBS, pH = 7.4, mixed with BHT-TCA (Trichltoracetic acid, Buthylhydroxytoluen) solution (1‰ w/v BHT dissolved in 20% TCA), centrifuged at 1000 g for 35 min and finally mixed with 0.5 N HCl and 120 mM TBA (Thiobarbituric acid) in 26 mM Tris and heated in water bath at 80°C for 10 min. After cooling, the absorbance of the resulting chromophore was measured at 532 nm. MDA levels were determined by using an extinction coefficient for MDA-TBA complex of 1.56 10^5^ M^-1^ cm^-1^.

### Thiol groups measurement

Total concentration of thiol groups (−SH) was performed according to Ellman’s method [27]. Briefly, aliquots from liver tissue was mixed with 100 μl of 10% SDS and 800 μl of 10 mM phosphate buffer (pH 8) and the absorbance was measured at 412 nm (A0). Then, 100 μl of DTNB were added and incubated at 37°C during 60 min. After incubation the absorbance of the sample was measured at 412 nm (A1). The thiol groups concentration was calculated from A1-A0 subtraction using a molar extinction coefficient of 13.6 × 103 M-1 × cm-1. Results were expressed as nmol of thiol groups per mg of protein.

### Antioxidant enzyme activities assays

The activity of superoxide dismutase (SOD) was assessed by the spectrophotometric method of Misra and Fridovich [28]. Mn-SOD activity was measured in the presence of 2.0 mM sodium cyanide, an inhibitor of Cu, Zn-SOD [29]. Catalase (CAT) activity was measured by the method of Aebi [30].

### Protein determination

Protein concentration was determined according to Bradford method [31]. Serum albumin was used as standard.

### Functional and metabolic parameters

#### Glycaemia assays

Glucose was measured by the glucose oxidase and peroxidase using quinoneimine as a chromogen. The amount of plasma glucose is related to amount of quinoneimine, which is measured spectrophotometrically at 505 nm [32].

#### Assessment of liver function

Plasma aspartate aminotransferase (AST), alanine aminotransferase (ALT), phosphatase alcaline (PAL), lactate dehydrogenase (LDH) were measured using commercially available diagnostic kits (Biomaghreb, Ariana, TN).

#### Assessment of renal function

Plasma urea, creatinine, uric acid and albumin analyses were performed using commercially available diagnostic kits (Biomaghreb, Ariana, TN).

#### Metabolic parameters

Plasma total cholesterol (TC), low-density lipoprotein-cholesterol (LDL), high-density lipoprotein-cholesterol (HDL) and triglyceride (TG) concentrations were measured using commercially available diagnostic kits supplied by Randox laboratories (Ardmore, Northern Ireland, UK).

### Statistical analyses

Data were analyzed by unpaired Student *t*-test and are expressed as mean ± standard deviation. Data are representative of 12 independent experiments. All statistical tests were two-tailed, and a *p* value of 0.05 or less was considered significant.

## Results

### Chemical composition of the lavender essential oils

The results obtained by GC–MS analyses of the *L. stoechas* essential oils (extraction yield = 0.05%) are presented in Table 1. Twenty two compounds were identified. The principal compounds detected are: D-Fenchone (29.28%), α-pinene (23.18%), Camphor (15.97%), Camphene (7.83%), Eucapur (3.29%), and Limonene, (2.71%) Linalool, (2.01%) Endobornyl Acetate (1.03%). The essential oils also contained lower percentages of Tricyclene, Cymene, Delta-Cadinene, Selina-3,7(11)-diene.

**Table 1:** Phytochemical compositions of L. Stoechas essential oils are presented in Table 1

**No**	**Components**	**IR**	**Compositions (%)**
**1**	Tricyclene	6.137	0.51
**2**	α-pinene	6.720	23.18
**3**	Camphene	7.310	7.83
**4**	β-Phellandrene	8.500	0.10
**5**	β -Pinene	8.626	0.12
**6**	Delta 3-Carene	10.554	0.11
**7**	Cymene	11.504	0.72
**8**	Limonene	11.807	2.71
**9**	Eucapur	11.893	3.29
**10**	D-Fenchone	15.835	29.28
**11**	Linalool	16.785	2.01
**12**	Camphor	19.526	15.97
**13**	Myrtenol	23.228	0.26
**14**	Endobornyl acetate	29.122	1.03
**15**	Aromad endrene	33.991	0.28
**16**	α -Copaene	34.809	0.28
**17**	Caryophyllene	37.436	0.26
**18**	β -Selinene	41.533	0.26
**19**	Delta-Cadinene	43.953	0.67
**20**	α -Elemene	44.434	0.12
**21**	Selina-3,7(11)-diene	44.817	0.85
**22**	Delta-gurjunene	47.730	0.20

Twenty two compounds were identified in the essential oils as a result of GC–MS analyze.

*IR* Retention index.

### Antioxidant capacity of the lavender essential oils

**Figure 1:**
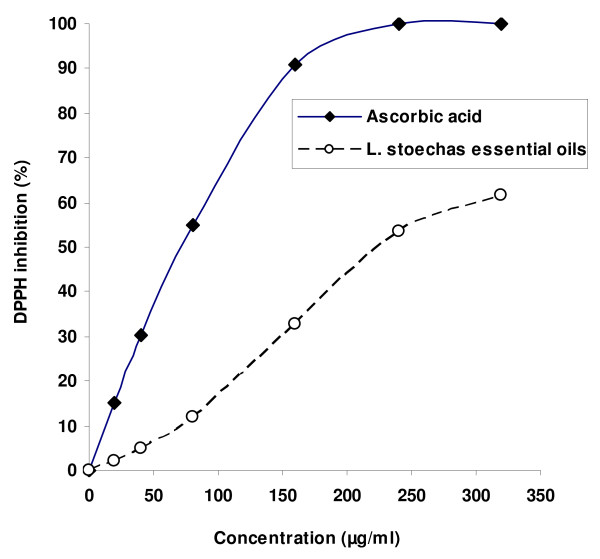
**Free radical-scavenging activity of *Lavandula stoechas *** **essential oils and ascorbic acid on 2,2-diphenyl-1-picrylhydrazyl (DPPH).**

Concerning the antioxidant capacity, we showed in Figure 1 that the radical-scavenging activity (RSA) of *L. stoechas* essential oils and ascorbic acid against DPPH radical increased significantly in a dose-dependant manner. However, *L. stoechas* essential oils showed an important RSA (EC50 = 221.43 μg/mL) but lesser than ascorbic acid (EC50 = 87.57 μg/mL) used as reference molecule.

### Body and relative organs weights

The weight gain has significantly decreased in alloxan-induced diabetic rats compared to healthy non diabetic animals. Subacute Lavender essential oils administration prevented this decline and protected also against alloxan-induced increase hepatic and renal relative weights (Table 2).

**Table 2:** **Subacute effect of *Lavandula stoechas *** **essential oils on acute aloxan-induced changes body weight as well as liver and kidney relative weights**

	**Healthy control**	**Diabetic control**	**Healthy + essential oils**	**Diabetic + essential oils**
**Initial body weight (g)**	227 ± 2.8	228 ± 2.3	230 ± 2.6	227 ± 2.9
**Final body weight (g)**	241 ± 2.7	227 ± 2.4^**a**^	244 ± 2.4^**b**^	231 ± 3.1^**b**^
**Liver relative weight (mg/g b.w.)**	33.42 ± 3.91	62. 13 ± 2.81^**a**^	36.83 ± 3.45^**b**^	39.51 **± 2.27**^**b**^
**Kidney relative weight (mg/g b.w.)**	8.7 ± 0.2	11.13 ± 0.71^**a**^	8.36 ± 0.94^**b**^	7.89 ± 0.4**9**^**b**^

Animals were treated during 15 days with *Lavandula stoechas* essential oils (50 mg/kg *b.w., i.p.)* or vehicle (NaCl 0.9%) seven days after diabetic induction with alloxan at 220 mg/kg *b.w.*. Assays were carried out in triplicate.

^**a**^*p* < 0.05 compared to “Healthy Control” group and ^**b**^*p* < 0.05 compared to “Diabetic Control” group.

### Blood glucose level

Significant increase in blood glucose level was observed in alloxan-induced diabetic rats compared to healthy non-diabetic animals (Table 3). Lavender essential oils treatment corrected significantly this decrease, while essential oils alone had no significant effect.

**Table 3:** **Subacute effect of *Lavandula stoechas *** **essential oils on acute aloxan-induced changes in liver and kidney functions**

	**Glucose**	**ALT**	**AST**	**ALP**	**LDH**	**Albumin**	**Urea**	**Uric acid**	**Creatinin **
	**(mM)**	**(UI/L)**	**(UI/L)**	**(UI/L)**	**(U/L)**	**(g/dl)**	**(mmol/L)**	**(mmol/L)**	**(μmol/L)**
**Healthy control**	6.65 ± 0.66	23 ± 1.59	39 ± 2.82	129 ± 7.1	938 ± 16	4.72 ± 0.93	7.64 ± 0.38	0.28 ± 0.01	114 ± 6.7
**Diabetic control**	15.29 ± 1.1^**a**^	63 ± 1.38^**a**^	68 ± 2.13^**a**^	179 ± 6.5^**a**^	1246 ± 24^**a**^	3.15 ± 0.96^**a**^	10.8 ± 0.40^**a**^	0.13 ± 0.02^**a**^	163 ± 5.9^**a**^
**Healthy + essential oils**	5.17 ± 0.6^**b**^	21 ± 1.75^**b**^	37 ± 3.63^**b**^	148 ± 6.8^**b**^	916 ± 13^**b**^	5.34 ± 0.30^**b**^	7.14 ± 0.35^**b**^	0.28 ± 0.01^**b**^	126 ± 6.3^**b**^
**Diabetic + essential oils**	7.81 ± 0.71^**b**^	32 ± 1.37^**b**^	43 ± 2.51^**b**^	155 ± 9.6	1095 ± 38^**b**^	4.13 ± 0.35^**b**^	7.21 ± 0.89	0.29 ± 0.01^**b**^	119 ± 8.4^**b**^

Animals were treated during 15 days with *Lavandula stoechas* essential oils (50 mg/kg *b.w., i.p.)* or vehicle (NaCl 0.9%) seven days after diabetic induction with alloxan at 220 mg/kg *b.w.*. Assays were carried out in triplicate.

^**a**^*p* < 0.05 compared to “Healthy Control” group and ^**b**^*p* < 0.05 compared to “Diabetic Control” group.

### Effect of lavender essential oils on hepatic and renal lipid peroxidation and thiol groups content

**Figure 2:**
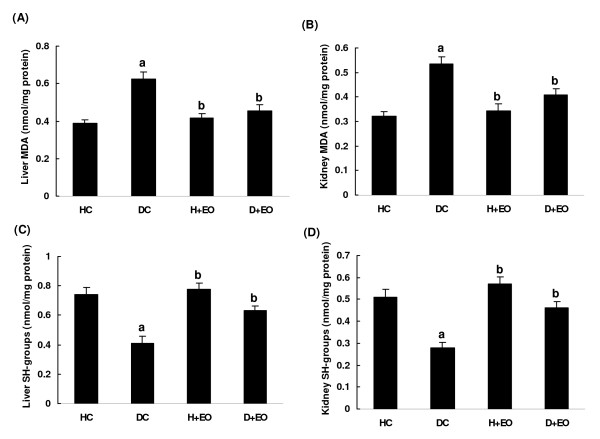
**Subacute effect of *Lavandula stoechas *essential oils on acute aloxan-induced changes in liver and kidney MDA (A and B) and (−SH) groups (C and D) levels.** Animals were treated during 15 days with *Lavandula stoechas* essential oils (50 mg/kg *b.w., i.p.)* or vehicle (NaCl 0.9%) sevan days after diabetic induction with alloxan at 220 mg/kg *b.w.*. Assays were carried out in triplicate. **HC**: Healthy Control; **DC**: Diabetic Control; **H + EO**: Healthy + Essential Oils; **D + EO**: Diabetic + Essential Oils. **“a”**: *p* < 0.05 compared to “Healthy Control” group and “**b”**: *p* < 0.05 compared to “Diabetic Control” group.

Alloxan-induced diabetes significantly increased liver and kidney MDA level as index of lipid peroxidation and decreased thiol groups content compared to healthy non-diabetic group (Figure 2A,B,C and D). Treatment with lavender essential oils (50 mg/kg *b.w., i.p.*) protected against the lipoperoxidation as well as the decrease of (−SH) groups level induced by alloxan treatment. Lavender essential oils alone had no effect on lipid peroxidation.

### Effect of lavender essential oils on liver and kidney antioxidant enzyme activities

**Figure 3:**
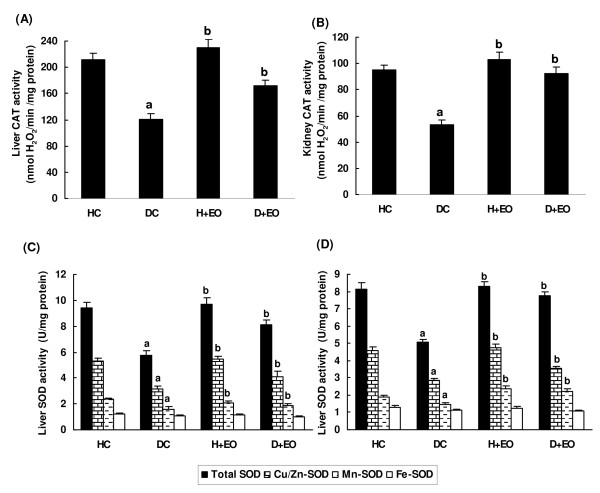
**Subacute effect of *Lavandula stoechas *essential oils on acute aloxan-induced changes in liver and kidney CAT (A and B), Total SOD, Cu/Zn-SOD, Mn-SOD and Fe-SOD (C and D).** Animals were treated during 15 days with *Lavandula stoechas* essential oils (50 mg/kg *b.w., i.p.)* or vehicle (NaCl 0.9%) sevan days after diabetic induction with alloxan at 220 mg/kg *b.w.*. Assays were carried out in triplicate. **HC**: Healthy Control; **DC**: Diabetic Control; **H + EO**: Healthy + Essential Oils; **D + EO**: Diabetic + Essential Oils. **“a”**: *p* < 0.05 compared to “Healthy Control” group and “**b”**: *p* < 0.05 compared to “Diabetic Control” group.

We further looked at the effects of alloxan and Lavender essential oils treatment on hepatic and renal antioxidant enzymes activities. Results presented in the Figure 3A,B,C and D, showed that alloxan administration significantly decreased hepatic and renal antioxidant enzyme activities as CAT, total SOD, cu/zn-SOD and Mn-SOD. Lavender essential oils treatment significantly reversed alloxan-induced antioxidant enzymes depletion.

### Effect of lavender essential oils on liver and kidney functions

The exposure of rats to alloxan led to liver dysfunctions as indicated by the of AST, ALT, ALP and LDH activities (Table 3). Concerning kidney function, we showed that alloxan exposure induced an increase of plasma creatinin and urea levels as well as a decrease of albumin and acid uric contents (Tables 2 and 3). Treatment with *L. stoechas* essential oils (50 mg/kg *b.w., i.p.*) significantly protected against hepatic and renal dysfunctions induced by alloxan treatment.

### Effect of lavender essential oils on lipid metabolic parameters

Table 4 showed that alloxan exposure of rat induced considerable metabolic disorders. Indeed, alloxan treatment of rats significantly increased the TG, TC and LDL plasma contents and decreased the HDL level. Importantly, we showed that *L. stoechas* essential oils supplementation significantly protected against the disturbance of lipid metabolic parameters induced by alloxan.

**Table 4:** **Subacute effect of *Lavandula stoechas *** **essential oils on acute aloxan-induced changes in lipid metabolic parameters**

	**TG (mg/ml)**	**TC (mg/ml)**	**HDL (mg/ml)**	**LDL (mg/ml)**	**TG/HDL**	**TC/HDL**
**Healthy control**	0.66 ± 0.02	0.72 ± 0.02	0.19 ± 0.02	0.21 ± 0.03	3.47	3.78
**Diabetic control**	1.28 ± 0.07^**a**^	0.87 ± 0.04^**a**^	0.13 ± 0.02^**a**^	0.34 ± 0.02^**a**^	9.84^**a**^	6.29^**a**^
**Healthy + essential oils**	0.68 ± 0.16^**b**^	0.68 ± 0.03^**b**^	0.14 ± 0.01^**b**^	0.24 ± 0.02^**b**^	4.85^**b**^	4.85^**b**^
**Diabetic + essential oils**	0.86 ± 0.12^**b**^	0.74 ± 0.04^**b**^	0.16 ± 0.02^**b**^	0.27 ± 0.03^**b**^	5.37^**b**^	4.62^**b**^

Animals were treated during 15 days with *Lavandula stoechas* essential oils (50 mg/kg *b.w., i.p.)* or vehicle (NaCl 0.9%) seven days after diabetic induction with alloxan at 220 mg/kg *b.w.*. Assays were carried out in triplicate.

^**a**^*p* < 0.05 compared to “Healthy Control” group and ^**b**^*p* < 0.05 compared to “Diabetic Control” group.

## Discussion

It was noteworthy that the composition of *L. stoechas* essential oils in North Africa was in partial agreement with the previous report [33]. Thus, 8-cineole constituted the major constituent of *Lavandula* essential oils in agreement with previous report [33]. In contrast, other compounds as p-cymen-8-ol, pinocarvone and α-terpineol and α -terpinen-7-al and fenchone, were not detected in our sample, but were found in other report [34]. Sabinene, which was present at low concentration (1.4%) in the previous report [34], represented one of the major constituents in our sample (13.89%). These changes in the essential oils composition are mainly related to the climatic and geographic conditions as well as genetic differences [33]. However, several investigations on the essential oils of various Lavandula species [33,35-37] showed that 1, 8- cineole, linalool, linalyl acetate, fenchone and *β*-phellandrene are the characteristic compounds of these plants.

On the other hand, using the DPPH radical-scavenging assay we showed that *L. stoechas* essential oils presented a higher scavenging capacity which may be related to the presence of phenolic compounds in accord with previous reports [33-37]. However, this antioxidant capacity remained lower than that of ascorbic acid.

Alloxan administration produced diabetes status by destruction of pancreatic β-cells [38] with changes in metabolic variables as well as kidney and liver functions. We firstly found that alloxan injection decreased body weight gain and increased kidney and liver relative weights. We also showed that alloxan administration increased glycaemia, cholesterol, triglycerides, urea, uric acid, creatinine, AST, and ALT levels. However, in the alloxan-induced diabetes mellitus, the rise in blood glucose is also accompanied by an increase in plasma cholesterol, triglycerides and urea [39,40].

The diabetogenic effects of alloxan are partly attributed to the specific cytotoxic action mediated by reactive oxygen species generation leading to the damage of large number of β-cells accompanied by a decrease in endogenous insulin release. However, alloxan-administered rats became hyperglycaemic in a short period of time, followed by a hepatic glucose overproduction [41]. More importantly, we have shown that *L. stoechas* essential oils decreased blood glucose in alloxan-diabetic rats. *L. stoechas* oils may exert their antihyperglycaemic effect by potentiating plasma insulin action, secretion or its release from bound form [42]. In diabetic status, lipoprotein lipase is not activated due to insulin deficiency resulting in hypertriglyceridemia and hypertriglyceridemia. This is in agreement with the fact that the glycemia level is the major determinant of total and very low-density lipoprotein cholesterol concentrations [43]. Our data indicated that alloxan-induced diabetic rats presented high levels of plasma urea, uric acid and creatinine, which are considered as significant markers of renal function [44,45]. Treatment with *L. stoechas* essential oils reversed all these parameters to near control levels. The increase of plasma AST and ALT activities indicated that diabetes may induce hepatic dysfunction as supported by previously findings showing a necrotic liver [46]. Therefore, the increase of transaminase activities in plasma may be mainly due to the leakage of these enzymes from the liver [47]. On the other hand, treatment of the alloxan-diabetic rats with *L. stoechas* essential oils restored the transaminase activities. These results are in line with those obtained by Ohaeri [48] and illustrate the hepatoprotective effects of lavender against alloxan-induced toxicity.

More importantly, *L. stoechas* supplementation, protected against alloxan-induced decrease of plasma uric acid concentration as one of the major endogenous water-soluble antioxidants [49], as well as tissue malondialdehyde increase, as marker of lipid peroxidation [50]. We also showed that alloxan administration decreased antioxidant enzymes activities such as SOD and CAT. However, these enzymes are known to be inhibited in diabetic model as a result of non-enzymatic glycosylation and oxidation [51]. These results corroborate previous reports [51,52] indicating the interaction between diabetes and ROS generation. Subacute treatment of alloxn-treted rats with *L. stoechas* essential oils increased the activities of these enzymes, which might be due to decreased oxidative stress. Previous studies have well shown the richness of lavender extracts as well as essential oils in phenolic compounds [53]. Theses molecules are the primal source of antioxidant ability of this plant, by scavenging free radicals as hydroxyl radical (OH^.^) which is the major cause of lipid peroxidation [54]. However, diabetic status is accompanied by high oxidative stress due to persistent and chronic hyperglycaemia leading to the depletion of antioxidant defense system and free radicals generation [55]. Hydroxyl radical (OH^.^) reacts with all biological substances and the most susceptible ones are polyunsaturated fatty acids leading to lipid peroxidation [56]. Indeed, increased lipoperoxidation impairs membrane function by decreasing membrane fluidity and changing the activity of membrane-bound enzymes and receptors [57].

In conclusion, our data clearly demonstrated the protective effects of the *lavandula stoechas* essential oils against diabetes and oxidative stress induced by alloxan treatment in rat. Lavender essential oils decreased kidney and hepatic injuries mainly through their antioxidant properties and played a major role as hepato- and nephroprotector products.

## Abbreviations

ALT: Alanine aminotransferase; AST: Aspartate aminotransferase; BSA: Bovine serum albumin; CAT: Catalase; HDL-C: HDL-cholesterol; LDL-C: LDL-Cholesterol; MDA: Malondialdehyde; PAL: Phosphatase alkalines; SOD: Superoxide dismutase; TC: Total-cholesterol; TG: Triglyceride; LPO: Lipoperoxidation.

## Competing interests

The authors declare that they have no competing interests.

## Authors’ contributions

HS and SS performed experiment s and wrote the first version of the manuscript. KR, AS, NG and MS participated in the design of the study, data analysis, and editing of the manuscript. All authors read and approved the final version of the manuscript.
